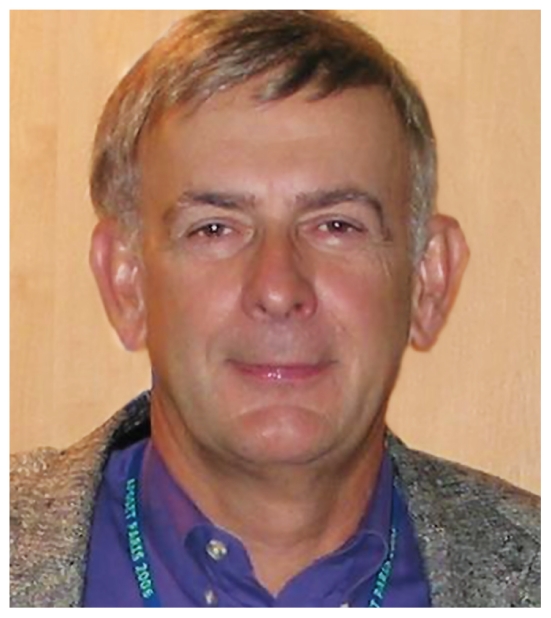# Larry L. Needham 1945**–**2010

**Published:** 2011-01

Larry L. Needham, a strong scientist and a staunch advocate for the use of exposure science in public health, passed away on 23 October 2010 after a 2-year battle with renal cell carcinoma. He will be remembered as a scientist with great vision who had the ability to develop and sustain impactful research programs that provided a strong underpinning for furthering exposure science. These programs served as the bedrock of environmental health throughout the past several decades.

Needham graduated from Middle Tennessee College and earned his doctorate in Organic Chemistry from the University of Georgia (UGA). After two postdoctoral fellowships at Vanderbilt University and UGA, he spent a short time as an assistant professor at Auburn University and as a chemist at General Electric. Needham then joined the Centers for Disease Control and Prevention (CDC) in 1976. Throughout his 34-year career in environmental health, Needham always approached his research with dedication and creativity, managing to develop the premier laboratory for biological monitoring of exposure and to sustain its growth and productivity by mentoring scientists in the field. Furthermore, he continued to broaden his knowledge and experience base to envelop other areas of exposure analysis. Needham’s contributions were significant and substantial, and only ceased when he was too critically ill to work. His contributions had a profound influence on strategic planning and on specific programs and issues. He developed innovative and highly refined approaches for assessing exposure to environmental toxicants. Some specific examples of his most noted scientific contributions are mentioned below.

The chemical terrorism laboratory at the CDC has its roots with Needham. In the mid-1990s, Needham recognized the potential threat of chemical warfare agents, such as nerve agents, and implemented a program to develop and validate methodology to measure metabolites of the five most common nerve agents: sarin, soman, tabun, cyclosarin, and VX. He was responsible for the laboratory developing methods for monitoring mustard gas, trichothecene mycotoxins, and marine toxins, and the initial setup of the Laboratory Response Network for chemical terrorism was conducted under his direction. Subsequently, several chemical terrorism biomonitoring methods were transferred to state health departments. Had it not been for Needham’s foresight, the capacity of the CDC and state health departments to respond to chemical terrorism events would not exist, and many critical response needs would not be available.

Needham also successfully grew the CDC pesticide program to be one of the most comprehensive in the world for assessing human exposure. In this effort, he was able to identify which pesticides would be banned or restricted and which pesticides would eventually replace them. Including the now-banned organochlorine pesticides, Needham’s laboratory routinely measured more than 150 different pesticides in biological media. His laboratory was sought out to collaborate in many critical health-related studies, which still serve as a basis for national policy.

Needham was a world-renowned researcher in environmental health studies of persistent organic pollutants (POPs). Beginning with research in the early 1980s that showed that only a fraction of the Vietnam veterans exposed to Agent Orange—and not the ones predicted by exposure indices—had elevated levels of dioxin, Needham was a primary player and leader in this field of research. He conferred with the United Nations Environment Programme on POPs on numerous occasions. Needham was a permanent member of the International Advisory Board for the annual dioxin meetings and was editor of *Chemosphere: Persistent Organic Pollutants and Dioxins*. He was also on the Editorial Review Board of *Environmental Health Perspectives*. In 2005 Needham presented before National Academy of Science panels on dioxins and on biomonitoring.

Few people have been as intimately involved in responding to the chemical emergencies that surface throughout the world as Needham. He led teams of scientists in quickly identifying the chemical etiologic agent of mass deaths in many poisoning situations. For example, he was instrumental in determining that a parathion-contaminated truck was responsible for contaminated flour in Sierra Leone. The contaminated flour was used to make bread products, which caused many deaths after ingestion. After identifying the contamination source, the flour and bread products were quickly discarded, and the deaths ceased. Furthermore, Needham led the investigation into a childhood epidemic of renal failure that eventually killed more than 80 children in Haiti. Within 36 hr, he identified diethylene glycol in acetaminophen preparations that the children were given to reduce fever. He subsequently found that the diethylene glycol had been mistakenly substituted for glycerin in the preparation. His quick identification of the etiologic agent combined with the removal of the medication from stores, as well as a broad-based media campaign to alert Haitians to the dangers of the preparation, immediately halted the mysterious deaths. On a national level, Needham provided the primary laboratory response in a multiagency investigation of widespread illegal application of methyl parathion in several Midwestern and Southern states. He measured the primary metabolite of methyl parathion in over 16,000 urine samples to assist in prioritizing relocation and decontamination efforts. His contribution saved the government an estimated $70 million in unneeded evacuations and decontamination costs.

Today’s leaders have a responsibility to foster scientific and professional growth among their charges. eedham did this very effectively and selflessly. He saw new scientists as “diamonds in the rough” who could be polished to be the leaders of tomorrow. He worked with these individuals to build their scientific capabilities, and he led them along their professional path not only by direction but also by example. Needham routinely encouraged them to grow as scientists by giving them many professional opportunities, such as participating in training programs, presenting data at scientific meetings, and publishing original scientific work. He thoughtfully critiqued all areas of their job performance, trying to evoke their optimum performance. In essence, he took inexperienced scientists and transformed them into competent, confident future leaders of exposure science.

Needham clearly had a huge impact on environmental health issues. He published more than 350 peer-reviewed journal articles and received many prestigious awards, such as the CDC’s Supervisor of the Year, CDC’s Honor Award for Research—Operational, the Mackel Award, the U.S. Environmental Protection Agency Silver Medal for Superior Service, the Public Health Service (PHS) Special Recognition Award, the PHS Superior Service Award, the Health and Human Services Secretary’s Award for Distinguished Service, the International Society of Exposure Science (ISES) Mehlman Award for affecting policy, and the ISES Wesolowski Award for scientific excellence. Needham served as the federal co-chair of the Chemical Exposures Workgroup for the multiagency National Children’s Study and served as a councilor, president-elect, and president of ISES.

In his personal life, Needham was an avid golfer and tennis player, and he was very active in the Dahlonega Methodist Church. He spent the last several months of his life reconnecting with friends and family and doing the things he loved. Needham is survived by his wife, Doris Needham, his son and daughter-in-law, Lance and Desiree Needham, and a grandson, Loghan Needham.

Needham will be missed immensely by those who worked with him and learned from him. He will be remembered for his many contributions to science and sustained leadership in furthering the field of environmental health.

## Figures and Tables

**Figure:**